# Generative Language Models for Disease Treatment Recommendations: A Systematic Literature Review

**DOI:** 10.1007/s41666-026-00242-y

**Published:** 2026-08-03

**Authors:** Leonides Medeiros Neto, Maicon Herverton Lino Ferreira da Silva Barros, Kayo Henrique de Carvalho Monteiro, Estefani Pontes Simão, Sebastião Rogério da Silva Neto, Luis Filipe Silva de Vasconcelos, João Pedro Pimenta, Iago Richard Rodrigues, Patricia Takako Endo

**Affiliations:** 1 Programa de Pós-Graduação em Engenharia da Computação (PPGEC), Universidade de Pernambuco (UPE), Recife, PE Brazil; 2 Universidade Católica de Pernambuco (UNICAP), Recife, PE Brazil

**Keywords:** GenAI, LLM, Disease, Treatment, Recommendations

## Abstract

The rapid evolution of Generative Artificial Intelligence (GenAI) presents significant opportunities to transform healthcare, particularly in generating personalized treatment recommendations. This systematic literature review explores the current state of GenAI language models applications in various medical domains, assessing their effectiveness, applicability, and limitations. The review addresses nine specific research questions to understand the potential and challenges of integrating GenAI into clinical practice. We use the Preferred Reporting Items for Systematic Reviews and Meta-Analyses (PRISMA) guidelines. From a pool of 3237 studies, 42 were selected based on inclusion and exclusion criteria. These studies were analyzed to evaluate the use of generative language models, such as GPT-3 and GPT-4, in various medical domains including oncology, cardiovascular, gastrointestinal, and ophthalmological care. The analysis revealed that most GenAI applications in healthcare rely on general purpose LLMs to provide treatment recommendations. Fine-tuning with domain-specific data and prompt engineering were found to significantly improve output quality and reliability. However, persistent challenges include lack of clinical validation, ethical concerns such as bias, and issues related to transparency and regulatory compliance. While GenAI demonstrates strong potential to support clinical decision-making, real-world deployment remains limited due to unresolved ethical and validation issues. Future research should prioritize the development of interpretable, domain-specific models and rigorous clinical trials to ensure safe and effective integration into healthcare settings.

**Supplementary Information:**

The online version contains supplementary material available at https://doi.org/10.1007/s41666-026-00242-y.

## Introduction

The evolution of generative artificial intelligence (GenAI) technologies has brought a new era of possibilities across various sectors, including healthcare. The introduction of the transformer architecture by Vaswani et al. [1] made possible the rise of models like Generative Pre-trained Transformer (GPT) [2] and Bidirectional Encoder Representations from Transformers (BERT) [3], which revolutionized tasks in natural language processing (NLP), extending their utility to medical decision-making. These models, characterized by their advanced deep learning architectures and extensive training datasets, are increasingly being employed to assist healthcare professionals by providing personalized treatment recommendations, thus potentially improving patient outcomes and optimizing clinical workflows.

GenAI refers to a subset of artificial intelligence that focus on generating new content, ranging from text and images to music and code [4]. Considering the generation of text, while Large Language Models (LLMs) with high parameter count have garnered significant attention for their extensive capabilities, smaller models also play crucial roles in healthcare applications. They are typically smaller in scale compared, and can be more manageable in terms of computational demands and adaptability, making them suitable for applications where performance is crucial.

The integration of these generative models into medical practice is driven by their ability to understand and generate human-like text from vast amounts of unstructured medical data. This capability not only enhances information retrieval and data interpretation but also paves the way for personalized medicine by tailoring treatment recommendations to individual patient profiles. However, the deployment of such advanced technologies in sensitive environments like healthcare raises questions regarding their effectiveness, adaptability, and the ethical implications of their use.

Some recent works have already explored the usage of GenAI in recommending treatments for different diseases, highlighting its potential to improve the accuracy of clinical decisions. Giuffrè et al. [5] presented a systematic review that investigates the use of LLMs as medical chatbots in the management of digestive diseases. The research evaluates the accuracy, safety and clinical implications of these models in gastroenterology and hepatology, highlighting both their potential to assist in medical decision support and the challenges related to the reliability and interpretation of the generated responses. In contrast, Schwartz et al. [6] discussed the challenges and risks of using LLM in the diagnosis and treatment of infectious diseases, highlighting the lack of transparency and the potential for clinical errors.

These works underscore generative AI’s versatility but also highlight persistent challenges such as the need for rigorous validation, biases in training data, and integration complexities within clinical workflows. Addressing these challenges requires advancements in model design, greater transparency in AI decision-making, and robust regulatory frameworks to ensure the safe and ethical deployment of generative AI in healthcare.

This systematic literature review (SLR) aims to critically evaluate the current state of GenAI, focused on language models, in providing treatment recommendations across various medical conditions. The primary objectives are to understand their applicability in diverse medical domains and identify potential limitations and challenges in their real-world implementation. By analyzing peer-reviewed works and key findings, this review seeks to offer comprehensive insights into the capabilities of language models, their integration challenges, and the ethical considerations that must be navigated to harness their full potential in healthcare.

Through this exploration, the review will contribute to the ongoing discourse on AI in medicine, providing stakeholders with information to guide future research, development, and policy-making in the field. As AI continues to evolve, it is imperative to maintain a critical perspective on its role in healthcare to ensure it enhances the quality of patient care.

## Background

This SLR reviews published articles that evaluate or propose GenAI language models for generating medical treatment recommendations. These models, built on the Transformer architecture, have become the backbone of modern GenAI systems. As we analyzed these works, we identified key terms needing clarification to avoid misconceptions. This section provides background on LLMs, the Transformer architecture, and their adaptation for medical tasks to ensure a clearer understanding of the reviewed studies.

### The Transformer Architecture and the Rise of LLMs

Introduced by Vaswani et al. in 2017 [1] for language translation tasks, the transformer architecture quickly replaced traditional recurrence and convolutions—previously dominant in state-of-the-art models–by relying solely on self-attention mechanisms to capture contextual relationships across a sequence. This design enables highly parallel computation and better long-range dependency modeling, significantly improving efficiency and scalability, especially on graphics processing units (GPUs) hardware [1]. Since then, Transformer variants have been widely adopted in various language models: encoder-based models such as BERT, which excels in natural language understanding tasks [7], and decoder-based models like GPT, which are used for text generation [2]. The multi-head self-attention mechanism allows these models to focus on different aspects of input simultaneously, making them highly effective at generating coherent, context-aware text [1].

The scaling of transformer models gave rise to the term LLMs, referring to architectures trained on massive corpora that use publicly available internet data and contain hundreds of millions to hundreds of billions of parameters. Through prompting alone, LLMs are capable of zero-shot and few-shot learning, a phenomenon demonstrated by GPT-3 [8], where the model can perform several NLP tasks. These models exhibit general-purpose capabilities such as translation, question answering, summarization, commonsense reasoning, and can be adapted to domain-specific tasks like clinical question answering or treatment recommendations. For instance, GPT-4 achieved human-level performance across a wide range of standardized tests, suggesting a form of emergent intelligence arising from scale alone [9]. Since then, the race for artificial general intelligence has continued, with companies releasing bigger models in parameter count, both closed and open source [10]. However, despite their impressive abilities, LLMs are prone to what are known as hallucinations and may generate incorrect or misleading content without proper grounding or adaptation through fine-tuning. Several techniques aim to mitigate hallucinations, this remains an unsolved problem and an active area of research [11].

### Terminological Ambiguities In Model Classification

The field of GenAI in healthcare has seen inconsistent usage of terms like Small Language Models (SLMs), Medium Language Models (MLMs), and LLMs, reflecting a lack of standardization. These labels often rely on parameter count rather than the volume of pre-training data, making them relative and prone to shifting over time. For instance, the model proposed by Griewing et al. [12] (Mixtral-8x7B-Instruct) is labeled as an SLM. Although its parameter count might seem small (46.7B compared to state-of-the-art models with over 100B), it requires over 48GB of GPU memory to execute.

Similarly, BERT, once considered a large model with 340 million parameters–too large for consumer hardware at the time–is now viewed as a small model, as modern architectures like GPTs surpass 100 billion parameters [13]. Some state-of-the-art models now exceed 685 billion parameters [14]. In general, it might be better to use the term LLM to refer to any model pre-trained on a large corpus rather than to a strict parameter count that can be relative depending on the available resources.

Although larger models in parameter size tend to perform better for general language understanding tasks, size alone does not determine effectiveness, particularly for specialized tasks like disease treatment recommendation [15]. Research has shown that well-designed smaller models, trained on specialized, high-quality, domain-specific datasets, are not only computationally cheaper but can also outperform larger general-purpose models in specialized tasks, such as treatment recommendations [12, 16]. This highlights that model size alone is not the sole determinant of quality, and underscores the importance of dataset quality and task-specific adaptation.

### Adapting LLMs for Medical Tasks Through Fine-Tuning

A crucial element in the development of LLMs is the training pipeline, typically composed of two stages: pre-training and fine-tuning. During pre-training, models are exposed to vast amounts of unlabeled data and learn general language representations using self-supervised objectives such as next-token prediction (as in GPT) or masked word prediction (as in BERT) [2, 7]. This stage equips the model with broad linguistic and world-based knowledge. However, it is through post-training, commonly referred to as fine-tuning, that models are adapted to specific tasks, domains, or user intentions.

Fine-tuning can be supervised, using labeled datasets, or aligned via other techniques to ensure the model’s behavior is appropriate, reliable, and contextually relevant. In other words, fine-tuning is when a pre-trained model undergoes weight updates—whether full fine-tuning or parameter-efficient fine-tuning. We found this term can cause confusion, with some authors claiming they pre-trained a model, when in fact they used a pre-trained model and updated its weights, which is fine-tuning [16, 17].

LLMs can perform a range of core tasks that form the foundation of their utility in real-world applications, referred to as downstream tasks. In causal language modeling (CLM), the model generates text by predicting each token based on previous ones, enabling downstream tasks such as text completion, story generation and treatment plan recommendations. For question answering (QA) tasks, models generally rely on knowledge derived from fine-tuning on datasets composed of question-answer pairs, their internal parametric knowledge acquired during pre-training, or external documents to produce answers [8].

Instruction-following models respond to natural language commands with appropriate task completion, a capability enhanced by fine-tuning on prompt-response datasets [18]. In chat settings, models engage in multi-turn dialogues that require retention of context and conversational coherence—an increasingly important trait for applications like medical counseling or interactive decision support [19]. Instruction-following models can also be trained specifically for conversational interactions, creating what is referred to as AI assistants.

Supervised fine-tuning (SFT) is especially critical in aligning pre-trained models with domain-specific behaviors and ethical norms. Through SFT, a model learns from high-quality examples written by humans, improving its ability to follow instructions, remain factual, and avoid harmful outputs. For instance, OpenAI’s InstructGPT and later ChatGPT were fine-tuned using SFT, followed by reinforcement learning from human feedback (RLHF), dramatically enhancing their alignment with user expectations [18]. In medical domains, SFT may involve curating clinical dialogues or guideline-based responses to teach the model to provide accurate, empathetic, and context-sensitive recommendations, aligned with what a medical professional would respond.

Altogether, the evolution of GenAI through the Transformer architecture, the scaling of LLMs, and alignment techniques like SFT has enabled the development of flexible, powerful tools for language understanding and generation. Despite ambiguities in terminology and ongoing concerns about trustworthiness, these models hold significant promise in healthcare settings. This review focuses on evaluating how these models, through their architecture, training regimes, and capabilities, have been applied to treatment recommendations.

## Methodology

The objective of this SLR is to critically evaluate the application of GenAI in providing treatment recommendations across various medical conditions. To report this, we adhere to the Preferred Reporting Items for Systematic Reviews and Meta-Analyses (PRISMA) guidelines. Our review is composed of an initial search, and a supplementary search, reported in the following sections. The SLR seeks to address the following nine Research Questions (RQs):

- (RQ1) In which diseases have GenAI been applied for treatment recommendations?
- (RQ2) What types of GenAI models have been utilized for generating disease treatment recommendations?
- (RQ3) What types of treatments have been considered in GenAI-based recommendations?
- (RQ4) How is prompt engineering used in GenAI-based treatment recommendations?
- (RQ5) How are datasets used for GenAI training?
- (RQ6) How are GenAI models evaluated for treatment recommendations?
- (RQ7) What ethical and regulatory considerations arise from the use of GenAI in medical treatment recommendations?
- (RQ8) What are the common challenges and limitations associated with the implementation of GenAI in clinical practice?
- (RQ9) What future research directions are suggested by current works on GenAI for treatment recommendation?

### Initial Search

**Fig. 1:**
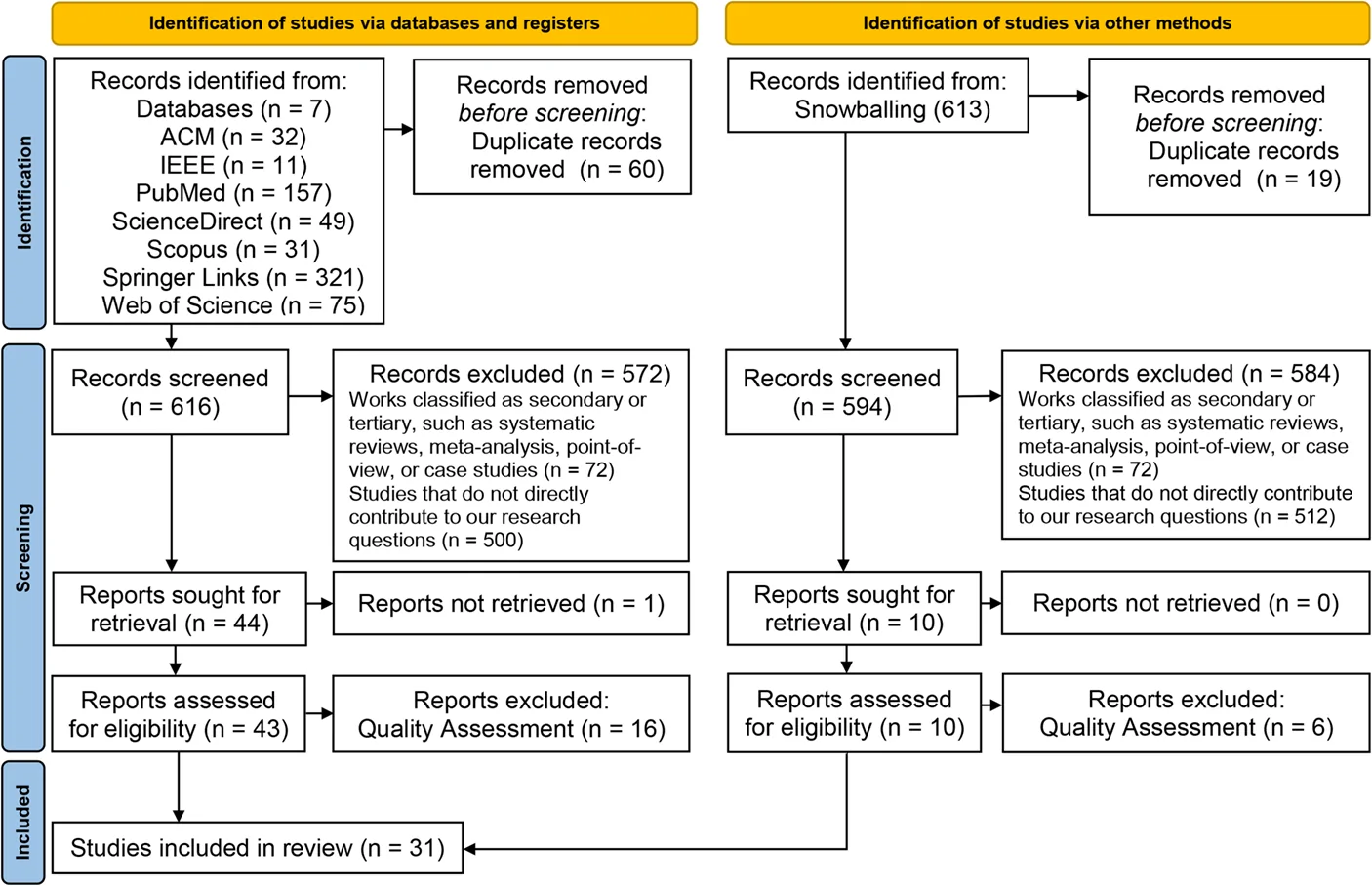
PRISMA with each step used to report our SLR. Flow chart created with Haddaway et al. [20]

This section details the search strategy for the initial search. Our initial database search follows PRISMA guidelines [20], and is presented in Fig. 1.

The search strategy encompassed a comprehensive selection of repositories that provide an extensive body of literature across various topics, including publications specifically related to computing and healthcare. The selected repositories include:

- ACM Digital Library
- IEEE Digital Library
- PubMed
- ScienceDirect
- Scopus
- Springer Link
- Web of Science

The search was conducted on 8 January 2025, the timespan covered by this search string was 2017 to 2024. To ensure a systematic and consistent retrieval of relevant works, a baseline search string was defined:

((“SLM” OR “MLM” OR “LLM”) OR ((“small” OR “medium” OR “large”) AND “language model”)) AND “recommendation” AND “disease treatment”

This search string required adaptation for each database to comply with their respective search syntax tool and indexing rules. The adjustments included variations in Boolean operators, field-specific queries, and term mapping to align with each platform’s query structure. These refinements ensured a precise and exhaustive retrieval of relevant literature while minimizing the inclusion of irrelevant works. The exact search strings used in each database are the following:

- **ACM:** ((“slm” OR “mlm” OR “llm”) OR ((“small” OR “medium” OR “large”) AND “language model” )) AND “recommendation” AND “disease treatment”
- **IEEE:** (“All Metadata”:llm OR “All Metadata”:slm OR “All Metadata”:mlm OR “All Metadata”:small language model OR “All Metadata”:medium language model OR “All Metadata”:large language model) AND (“All Metadata”:recommendation) AND (“All Metadata”:disease treatment)
- **PubMed:** ((((SLM) OR (MLM) OR (LLM)) OR (((small) OR (medium) OR (large)) AND (language model))) AND (recommendation) AND (disease treatment))
- **ScienceDirect:** (“llm” OR “slm” OR “mlm” OR (“large” OR “small” OR “medium” AND “language model”)) AND “recommendation” AND “disease treatment”
- **Scopus:** ((“slm” OR “mlm” OR “llm”) OR ((“small” OR “medium” OR “large”) AND “language model” )) AND “recommendation” AND “disease treatment”
- **Springer Link:** ((“slm” OR “mlm” OR “llm”) OR ((“small” OR “medium” OR “large”) AND “language models”)) AND “recommendation” AND “disease treatment”
- **Web of Science:** ALL=(((slm OR mlm OR llm) OR ((small OR medium OR large) AND language model )) AND recommendation AND disease treatment)

We expanded the database search with a single round of the snowballing technique, following guidelines proposed by Wohlin [21]. This technique can be used alongside database search to complement with relevant works that the search string might have missed [21], analyzing references and citing works from a start group (backward and forward snowballing). The start group was composed of the three most cited papers from the 31 works included via the initial database search, and Dai et al [16] selected for its exemplary best practices in their methodology. From the start group we identified 613 candidate papers, we screened them using the same methodology as the database search, described from subsection 3.3 to 3.5. After screening, we included 4 new works.

### Supplementary Search

**Fig. 2:**
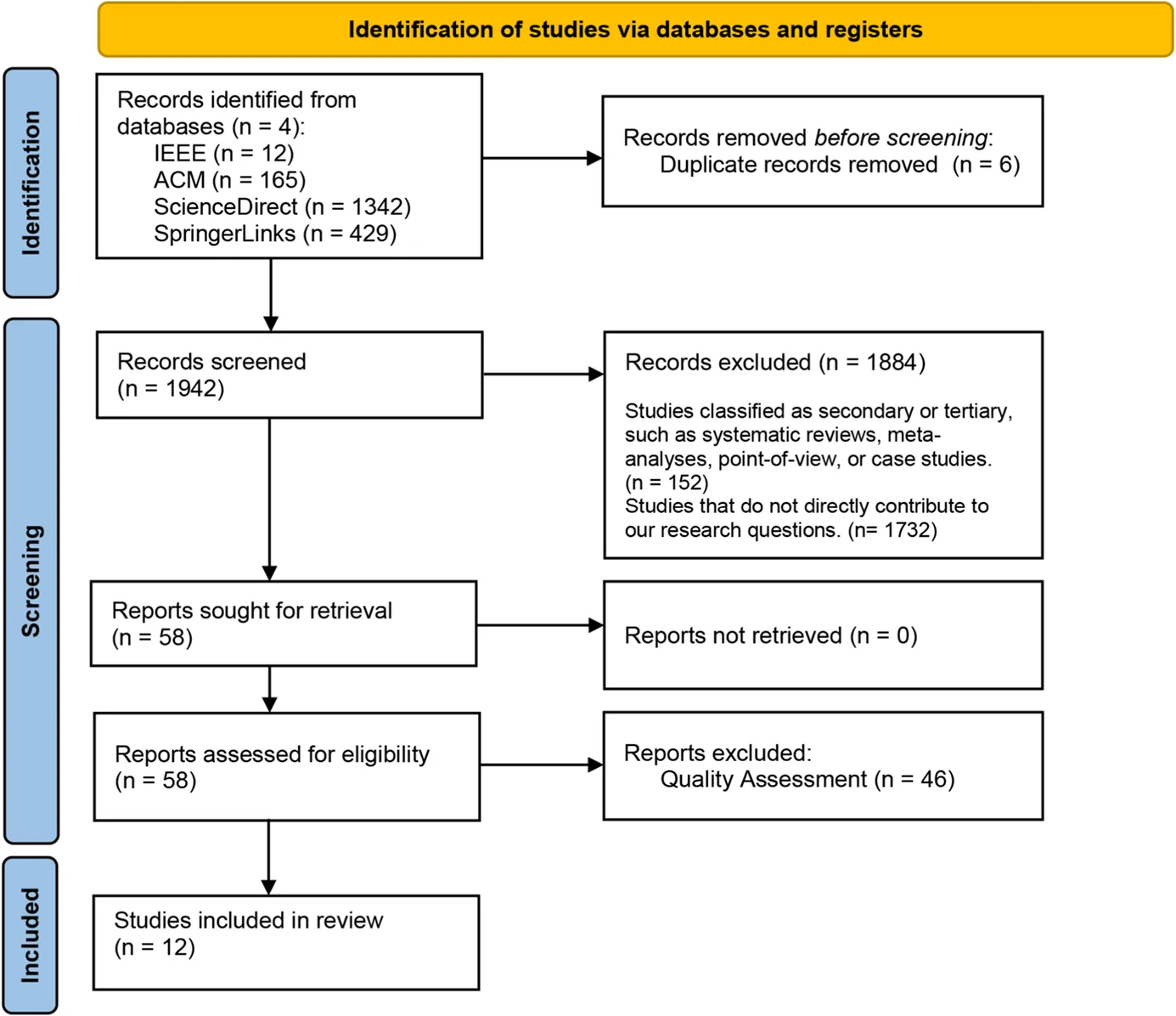
PRISMA reporting the supplementary search. Template provided by Haddaway et al. [20]

As part of the revision process, we further complement our search strategy with a supplementary database search. Our review benefits from a supplementary search by finding more relevant works, specially given the specific nature of the GenAI application we are studying. In addition, due to the rapid technological advancement in GenAI, in the process of writing this review new works were published, a supplementary search also strengthens the review by finding more recent relevant works. The PRISMA flow chart for the supplementary search is presented in Fig. 2.

We made several improvements in our supplementary search string to find more relevant works while balancing accuracy and precision. The improvements included revising the databases, adding more terms, synonyms, and boolean operations. With this search string we focus on original sources rather than relying on indexers, the databases used for the supplementary string are listed bellow:

- IEEE
- ACM
- ScienceDirect
- SpringerLink

The search was performed on 19 January 2026, and it spans works published from 2017 to 2025. The baseline for the supplementary string was crafted as follows:

(“language model” OR “generative model” OR “generative artificial intelligence”) AND (“clinical decision-making” OR “medical interventions” OR “clinical treatment”) AND (recommendation OR “decision support” OR assist*)

The baseline supplementary search string was adapted to some databases to follow the required syntax:

- **IEEE (Command search):** (“All Metadata”:“language model” OR “All Metadata”:“generative model” OR “All Metadata”:“generative artificial intelligence”) AND (“All Metadata”:“clinical decision-making” OR “All Metadata”:“medical interventions” OR “All Metadata”:“clinical treatment”) AND (“All Metadata”:recommendation OR “All Metadata”:“decision support” OR “All Metadata”:assist*) Field: all metadata, filters: journals, 2017-2025
- **ACM (advanced search):** (“language model” OR “generative model” OR “generative artificial intelligence”) AND (“clinical decision-making” OR “medical interventions” OR “clinical treatment”) AND (recommendation OR “decision support” OR assist*) Field: Anywhere, filters: range 2017-2025, content type: research article
- **ScienceDirect (advanced search):** (“language model” OR “generative model” OR “generative artificial intelligence”) AND (“clinical decision-making” OR “medical interventions” OR “clinical treatment”) AND (recommendation OR “decision support” OR assist) Does not support wild cards, Field: Find articles with these terms, filters: years 2017-225, article type: research article, language: English
- **SpringerLink (advanced search):** (“language model” OR “generative model” OR “generative artificial intelligence”) AND (“clinical decision-making” OR “medical interventions” OR “clinical treatment”) AND (recommendation OR “decision support” OR assist*)

### Study Selection

To define which works would be considered for inclusion or exclusion, we based our decision on the following criteria:

Inclusion criteria:

1. Primary works presented and available in full article format (except books, posters, tutorials or editorials)
2. Primary study exploring GenAI application for medical recommendation
3. Primary study published since 2017
4. Primary study written in English

Exclusion criteria:

1. Works classified as secondary or tertiary, such as systematic reviews, meta-analyses, point-of-view, or case works.
2. Works that do not directly contribute to our research questions.

In this step, each work was independently reviewed by at least two researchers tasked with determining whether it should proceed to the next stage. In the event of a disagreement or tie, a third researcher was assigned to make the final decision. During this phase, only the titles and abstracts were examined to assess relevance and suitability for inclusion in the review. We provide the full inclusion/exclusion decisions and metadata in Supplementary Material 2.

### Quality Assessment

After the initial classification of papers based on inclusion and exclusion criteria, the selected works proceeded to the quality assessment stage. This evaluation aimed to ensure the relevance and rigor of the selected works, following predefined criteria. The assessment consisted of the following three questions:

1. Are the objectives of the study clearly stated and related to the use of GenAI for medical recommendation?
2. Is the methodology described in sufficient detail?
3. Are the results clearly reported and supported by data (e.g., metrics)?

Each question was scored based on the following scale:

- **Yes** (2.0 points)
- **Partially** (1.0 point)
- **No** (0.0 points)

To qualify for the next phase, data extraction, a work needed to achieve an average quality assessment score of at least 3.0. This systematic evaluation ensured that only works with a well-defined scope, sufficiently detailed methodologies, and clearly presented results were included in the subsequent stage of the review.

### Data Extraction and Coding

In the data extraction phase, a systematic process was employed to identify and extract relevant information from the selected works to answer the research questions, such as the models used. This phase ensured that critical data points were captured consistently, allowing for a structured and comprehensive analysis.

To achieve this, we configured an online form that broke down our research questions into more detailed sub-questions. All selected works were reviewed by our researchers, who completed the form accordingly.

## Results

The initial search resulted in 31 works, 27 from the database search and 4 from the snowballing technique. The supplementary search resulted in 12 works included. In total, this SLR analyzes 42 relevant works. The following sections will address and discuss each research question defined earlier in our methodology.

### (RQ1) In which Diseases have GenAI been Applied for Treatment Recommendations?

**Fig. 3:**
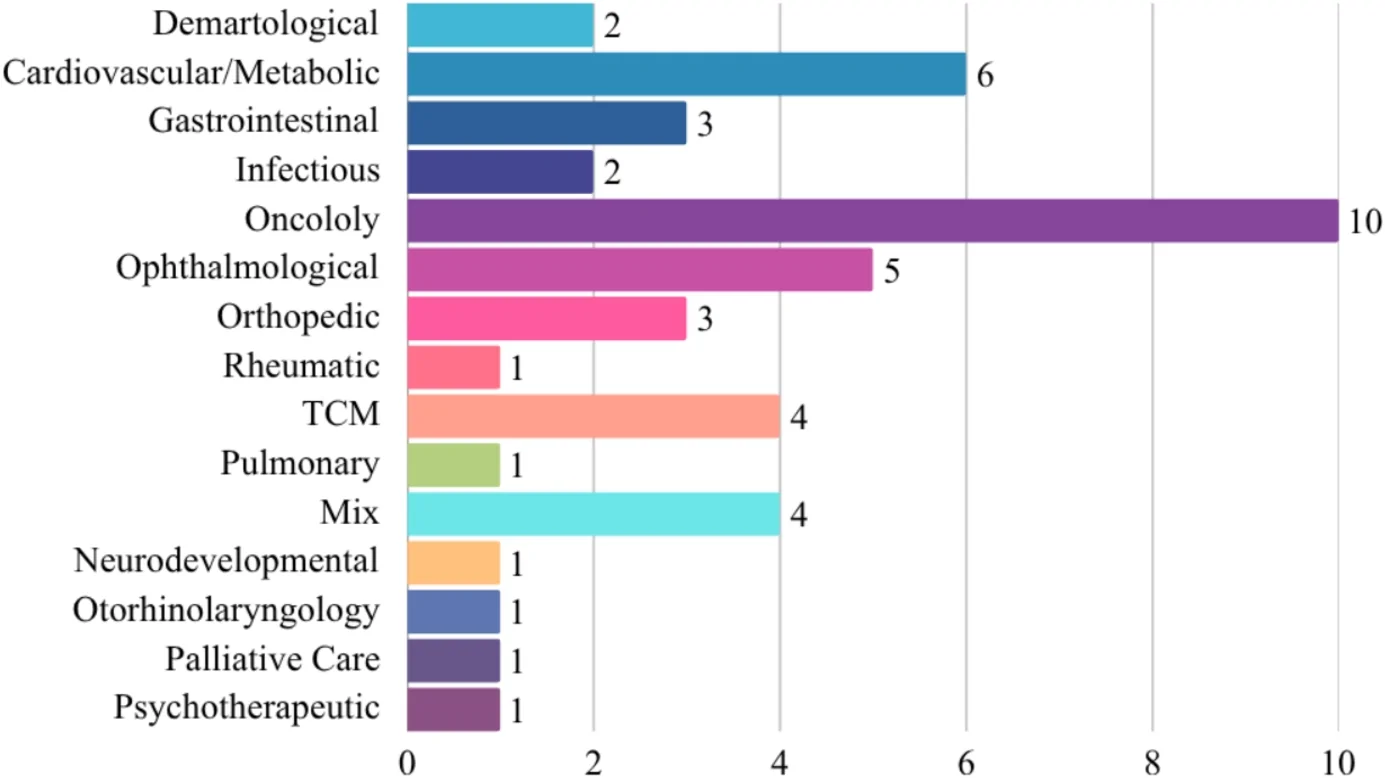
Number of works per diseases categories found in literature

The 42 works from the literature highlight the diverse applications of GenAI in medical treatment recommendations, covering nine distinct disease categories, as presented in Fig. 3 and detailed in Table 1.

**Table 1:** Most common diseases used by GenAI for treatment recommendation

Diseases	Works
Dermatological diseases	[22, 23]
Cardiovascular and metabolic diseases	[24–29]
Gastrointestinal diseases	[30–32]
Infectious diseases	[33, 34]
Oncology and cancer-related diseases	[12, 35–43]
Ophthalmological diseases	[15, 23, 44–46]
Orthopedic and musculoskeletal diseases	[47, 48]
Rheumatic and inflammatory diseases	[49]
Traditional Chinese Medicine (TCM)	[16, 17, 50, 51]
Pulmonary	[52]
Mixed diseases	[23, 53–56]
Neurodevelopmental disorder	[57]
Otorhinolaryngology	[58]
Palliative care	[59]
Psychotherapeutic	[60]

Ten studies are dedicated to oncology and cancer-related diseases, representing the highest number of works and highlighting the demand for personalized medical approaches. Oncology is followed by cardiovascular and metabolic, as well as ophthalmological diseases, with 6 and 5 related works, underscoring the importance of GenAI in addressing widespread conditions like heart disease and diabetes.

Additionally, the inclusion of works on Traditional Chinese Medicine (TCM) indicates a growing interest in leveraging GenAI beyond conventional western medical practices. According to the authors, TCM is based on a holistic health model, focusing on herbal medicines and their medical characteristics and effectiveness [16]. Furthermore, TCM uses dialectic methods that consider not only symptoms but also underlying patterns of energy imbalance in the body, such as imbalances in Yin and Yang energies. Diseases reflecting these imbalances, cardiovascular, respiratory, and gastrointestinal disorders, have been widely studied in TCM contexts [61]. Integration with conventional medicine can expand its therapeutic applications and improve clinical outcomes [17].

While most works focus on a single disease, some exceptions address multiple diseases. Liu et al. [17, 50] and Dai et al. [16] do not target a specific disease but aim to enhance the applicability of GenAI within the general framework of TCM. Li et al. [51] also focus on TCM, specifically on acupuncture, addressing conditions such as musculoskeletal pain, gastrointestinal and gynecological diseases, and pediatric ailments.

Wilhelm et al. [23] address 60 different diseases across three specialties: ophthalmology, dermatology, and orthopedics, including conditions like palmar fibromatosis, achilles tendon rupture, and more. Liu et al. [53] include a broad range of conditions such as campylobacter and related species infections, enteritis, gastrointestinal diseases, pancreatic cancer, liver cancer, digestive system diseases, retinal pathologies, and rheumatoid arthritis. Lastly, Neupane et al. [56] takes a more general approach across 10 diverse medical specialties, regardless of conditions.

Cancer-related diseases represent a significant portion of the reviewed works, with 10 works focusing on GenAI-assisted treatment recommendations [12, 35–43]. These works focus on various aspects of cancer treatment, including precision oncology, decision support for therapy selection, and the application of GenAI to enhance clinical decisions in oncology settings. Wang et al. [35] are focused on thyroid cancer, while 3 works focus on breat cancer [12, 38, 43]. Lammert et al. [36] apply GenAI for treatment recommendations in precision oncology. The system generated treatment recommendations for fictional oncology cases involving various types of cancer, including non-small cell lung cancer, breast cancer, melanoma, colorectal cancer, prostate cancer, pancreatic cancer, and glioblastoma, supporting personalized oncology care through molecularly targeted treatment suggestions. And Benary et al. [37] considers ten fictional cases representing seven different tumor types, with a focus on lung adenocarcinoma.

Six works address the use of GenAI in providing treatment recommendations for cardiovascular and metabolic diseases [24–27]. These works examine the role of GenAI in cardiovascular health management and its application in managing specific conditions such as diabetes and metabolic syndrome, highlighting its potential in both preventive and therapeutic contexts. Lautrup et al. [26] propose recommending treatments for cardiovascular diseases, including myocardial infarction, peripheral arterial disease, varicose veins, and general cardiovascular prevention, with a focus on delivering patient-specific health advice. Lee et al. [25] investigate bariatric and metabolic surgery, where GenAI supported clinical decision-making by recommending more appropriate surgical technique, perioperative management strategies, and postoperative care plans for patients with obesity.

Papastratis et al. [24] propose personalized meal plans for patients, specifically addressing obesity, cardiovascular diseases, and type 2 diabetes, by providing nutritional guidance tailored to individual patient profiles. Naja et al. [27] are focused on the nutritional management of type 2 diabetes and metabolic syndrome, offering dietary recommendations that address key components such as hypertension, hypertriglyceridemia, low HDL levels, hyperglycemia, and obesity, ensuring alignment with established clinical guidelines.

Ophthalmological diseases are also addressed by 5 works. Li et al. [44] and Choudhary et al. [45] are focused on diabetic retinopathy and diabetic macular edema, respectively, the remaining works in this category approach broader ophthalmological applications [15, 23, 46].

Truhn et al. and Shrestha et al. share a common theme in their application of GenAI to orthopedic and musculoskeletal diseases, despite their varied primary focuses. Truhn et al. [47] directly target orthopedic conditions, specifically knee and shoulder, utilizing GenAI to interpret magnetic resonance imaging (MRI) reports and generate treatment recommendations. Shrestha et al. [48] explore GenAI’s adherence to clinical guidelines for diagnosing and treating low back pain, while Wilhelm et al. [23] has a broader scope, including orthopedics as one of its key areas.

Three works revolve around improving management strategies for gastrointestinal diseases, with two specifically focusing on gastroesophageal reflux disease (GERD) [30, 32] and one on inflammatory bowel disease (IBD), focusing on colorectal dysplasia screening and endoscopic management [31].

Two works focus on dermatological diseases [22, 23]. Wilhelm et al. [23] are more broader in scope, including dermatological as one of its areas. While Iqbal et al. [22] investigate the reliability and accuracy of GenAI in generating medication recommendations for common skin diseases, as mentioned previously.

Labinsky et al. [49] report that GenAI models can generate first and second-line pharmacological treatment plans based on clinical vignettes. These recommendations were subsequently compared to those developed by a panel of rheumatology specialists and evaluated in terms of safety, adherence to clinical guidelines, medical appropriateness, completeness, and overall quality. They are the only work focused on rheumatic and inflammatory diseases.

GenAI has also been applied to provide treatment recommendations for bacterial meningitis, that is an infectious diseases, offering diagnostic and therapeutic suggestions based on clinical case scenarios. Fisch et al. [33] use GenAI models to identify central nervous system infections and recommend appropriate diagnostic procedures and empirical antibiotic regimens. However, variability in treatment accuracy highlights the need for further refinement to ensure reliability in real clinical applications.

Zakka et al. [54] cover multiple medical specialties, including cardiology, neurology, infectious diseases, pediatrics, gastroenterology, and anesthesia and critical care. The study introduces Almanac, a retrieval-augmented language model framework that provides accurate responses to clinical queries, offering evidence-based treatment options tailored to each specialty.

Lastly, one work proposes an LLM-based conversational AI therapist to analyze daily user behavior for psychotherapy interventions. Composed of different models for specific tasks the system is capable of applying Cognitive Behavioral Therapy (CBT) and Motivational Interviewing (MI), clinical practices generally administered by humans. The system use LLMs to actively steer the conversation to cover the psychotherapy needs identified as necessary by another model, they use GPTs 4 and 3.5 in their system [60].

Overall, oncology and cardiovascular disease emerge as the top areas where GenAI has been applied for treatment recommendations, likely due to their high global prevalence, large scale of data for training models, and the complexity of personalized treatment approaches. Furthermore, the appearance of TCM in several works also indicates a growing interest in leveraging GenAI for holistic and culturally tailored medical practices.

### (RQ2) What types of GenAI Models have been Utilized for Generating Disease Treatment Recommendations?

This section analyzes the models used in the works, either as a base for fine-tuning or directly for evaluation. Table 2 summarizes the models employed in the selected works. LLMs with closed weights, state-of-the-art at the time of writing of these works, dominate the landscape of GenAI applications for disease treatment recommendations, although open-weight models with lower parameter counts have emerged as alternatives due to their privacy when used locally and the possibility of fine-tuning to provide personalized medical recommendations [62].

**Table 2:** GenAI Models Utilized for Disease Treatment Recommendations

Model	Open	Works
Baichuan2-13B-Base	Yes	[17]
Baichuan2–7B-Chat	Yes	[16]
Bard*	No	[25, 32, 33]
Bing AI*	No	[25, 33, 45]
Bloomz	Yes	[23]
Copilot*	No	[32]
ChatGLM-6B	Yes	[50]
ChatGLM3-6B	Yes	[52]
Claude-2	No	[33]
Claude-3	No	[41]
Claude-instant-v1.0	No	[23]
Command-xlarge-nightly	No	[23]
DeepSeek-R1	Yes	[34, 55]
Gemini Pro*	No	[15, 36]
Gemini Pro 1.0	No	[59]
Gemini 1.5 flash	No	[28]
Gemma-2-9B	Yes	[58]
GPT-3.5	No	[12, 22–24, 27, 33, 39, 42, 48, 49, 57]
GPT-3.5 Turbo	No	[56, 60]
GPT-4	No	[12, 22–26, 30–33, 35, 37–39, 41, 57]
		[45, 47, 49, 51, 53, 54, 60]
GPT-4 Turbo Preview	No	[34, 42, 59]
GPT-4o	No	[29, 34, 40, 41, 43]
GPT-o1	No	[55]
Galactica	No	[37]
Llama-2	Yes	[44]
Llama-2-7B	Yes	[60]
Llama-2-7B-Chat	Yes	[46]
Llama-2-13B	Yes	[60]
Llama-2-70B	Yes	[33]
Llama-3-8B-Instruct	Yes	[58]
Mistral-7B-Instruct	Yes	[56, 59]
Mistral-Nemo-Instruct	Yes	[58]
Mixtral-8x7B	Yes	[12, 59]
Moonshot-v1-128k	No	[29]
PaLM-2	No	[33]
Perplexity*	No	[32, 37]
Qwen2.5-Instruct*	Yes	[34]

*Specific model not disclosed

The majority of works utilized proprietary models with closed weights, such as OpenAI’s GPT-4 and GPT-3.5 (Table 2). Three works used Gemini Pro, a platform by Google, with their best Gemini models. Other proprietary LLMs, such as Microsoft’s Copilot, and Anthropic’s Claude, have also been employed in some works. These models provide generative capabilities via online services and APIs from their respective companies. This trend highlights a current preference in the literature for high-performing but closed models, potentially limiting reproducibility and transparency. This preference may stem from the convenience of using powerful models, easy to access, without requiring local computational resources.

Models such as Baichuan2-7B-Chat [16], ChatGLM-6B [50], and LLaMA-7B fine-tuned [44], can be used locally and offer advantages due to privacy and controllability [62]. Unlike the aforementioned LLMs, these models are open-weight and can be deployed locally, fine-tuned with domain-specific data, enhancing their adaptability and reproducibility.

Recent works have explored the efficiency of open weights models in domain-specific applications, focusing on their optimization for specialized medical tasks. For instance, TCMChat [16] was built upon the Baichuan2-7B-Chat model that was already pre-trained and fine-tuned on a conversationalist dataset to behave as a chat. To adapt to domain knowledge, the authors further fine-tuned the model. Similarly, CPMI-ChatGLM [50] and Copd-ChatGLM [52] utilized ChatGLM-6B and ChatGLM3-6B used parameter-efficient fine-tuning techniques to improve treatment recommendations. Additionally, DeepDR-LLM [44] integrated a fine-tuned LLaMA-7B model to generate treatment recommendations. A fine-tuned Bloomz was used in one work [23], highlighting how fine-tuned models are used in medical recommendations. These open-weight models showcase the potential for tailored and transparent model development in healthcare contexts.

Lastly, Griewing et al. [12] found that the smaller open-weights model Mixtral-8x7B, when used with medical data, shows performance comparable to human evaluators and larger state-of-the-art models such as GPT-4 in breast cancer treatment recommendations. This suggests that smaller models can be adapted to improve result quality.

In summary, most studies relied on proprietary, closed-weight LLMs due to their strong performance and accessibility via APIs. However, a smaller subset of works has begun to explore open-weight alternatives, especially for local deployment and adaptation with domain-specific data, one study only focus on local models [58]. The selected works suggest that smaller, specialized models might be sufficient to generate high-quality results while also protecting personal data, as they can be used in a controlled environment locally [17]. These open models demonstrate promising potential for greater transparency, reproducibility, and customization in medical applications, marking a growing shift in the field.

### (RQ3) What Types of Treatments have been Considered in GenAI-Based Recommendations?

The included studies demonstrate that GenAI-based systems have been applied to a broad range of treatment recommendation domains. We categorized these recommendations into five types: (1) surgical, (2) pharmacological, (3) nutritional and lifestyle interventions, (4) TCM, and (5) clinical decision support or diagnostic assistance. Table 3 presents representative studies and the types of treatments considered, the remaining works fall outside of the categories.

**Table 3:** Types of Treatments Considered in GenAI-Based Recommendations

Treatment Type	Representative Studies and Summary	LLMs Used
Surgical	GERD [30, 32], bariatric [25], orthopedic [47], and breast cancer [12], head and neck cancer [39–41]. Validated against clinical decisions and guideline adherence.	GPT-3.5, GPT-4, GPT-4o, Claude 3, BC-SLM, Bard
Pharmacological	Dermatology [22], rheumatology [49], oncology [36, 37], infectious diseases [33], IBD [31].	GPT-4, BioMedLM, ChatGLM
Nutritional & Lifestyle	Diabetes and NCD management [24, 27], therapy recommendation [53], psychotherapy [60].	GPT-3.5, MED-ChatGPT, GPT-4
TCM	Acupuncture and herbal treatment via LLMs specialized in TCM [16, 17, 50, 51]. Models tailored for Eastern clinical treatments.	TCMChat, Lingdan, AcupunctureGPT
Decision Support & Diagnostics	Thyroid cancer [35], diabetes care [44], macular diseases [15], breast cancer [38], back pain [48], chronic obstructive pulmonary disease [52], and general-purpose tools [54].	GPT-4, ChatGLM3-6b, Almanac, DeepDR-LLM

Surgical interventions were addressed in multiple studies. GPT-based systems were evaluated for their ability to suggest surgical management for GERD [30, 32], bariatric surgery [25], and orthopedic cases using MRI data [47]. These models, particularly GPT-4, demonstrated encouraging alignment with clinical recommendations, especially when prompts were designed to mimic realistic patient scenarios. Griewing et al. [12] proposed a domain-specific SLM for breast cancer surgical decision-making, emphasizing transparency and expert validation.

Other studies examined LLMs’ ability to recommend drug-based (pharmacological) treatments across dermatology [22], rheumatology [49], oncology [36, 37], inflammatory bowel diseases [31], infectious diseases [33], and palliative care [59]. GPTs and other LLMs provided therapeutic suggestions that were often aligned with guidelines, though inconsistencies were reported in more complex cases [34].

GenAI was also used to generate dietary plans and lifestyle recommendations, especially for patients with chronic diseases. Three works explored dietary guidance for diabetes and metabolic syndrome [24, 27, 28], while Liu et al. [53] provided adjunctive lifestyle support. Although LLMs could provide general advice, concerns were raised regarding specificity, safety, and adherence to medical nutrition therapy guidelines.

We also found works that applied GenAI to recommend treatments rooted in TCM, including acupuncture, moxibustion, and herbal prescriptions. Studies using fine-tuned models like TCMChat [16], Lingdan [17], CPMI-ChatGLM [50], and AcupunctureGPT [51] showed promising results in replicating clinical recommendations aligned with TCM theory. These models outperformed general-purpose LLMs in TCM-specific tasks and were positively validated by domain experts.

Six studies evaluated LLMs as a tool for combined diagnostic reasoning and treatment planning. GPT-4 and similar models supported clinical decisions in areas such as thyroid cancer diagnosis [35], diabetic retinopathy management [44], and macular disease detection using vision-language models [15]. Others investigated alignment with international treatment guidelines in low back pain [48] and early breast cancer [38]. Tools such as Almanac [54] and DeepDR-LLM [44] demonstrated patient acceptance and high potential for real-world clinical integration.

### (RQ4) How is Prompt Engineering used in Genai-Based Treatment Recommendations?

Prompt engineering refers to the design and refinement of inputs in natural language to instruct and guide the generative models into generating answers. This practice is essential to help the model generate outputs aligned with the user’s intent [18], especially in healthcare where the outputs should be clinically relevant and accurate.

**Fig. 4:**
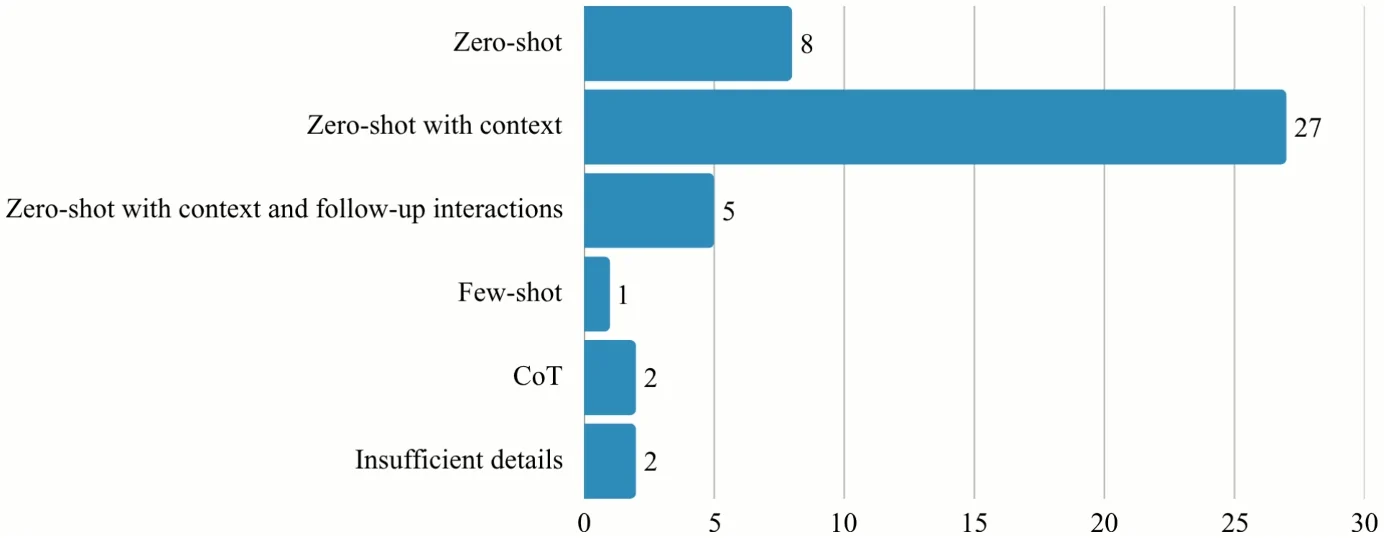
Number of works per type of prompt engineering

All reviewed works employ some form of prompt engineering. We classify the strategies into four categories: zero-shot, zero-shot with context, zero-shot with context and follow-up interactions, and CoT. We also indicate works that provide insufficient information to classify their approach. Figure 4 presents the number of works per type of prompt engineering strategy.

The majority of the works use some variation of zero-shot prompting, which is when the model is instructed to generate recommendations without explicit examples [8]. Eight works use zero-shot prompting, where the model receives only a single instruction or question without additional context. There are 27 works that employ zero-shot with context, incorporating additional information such as patient symptoms or disease background. Five works employ zero-shot with context and follow-up interactions, where the model receives context and additional clarifications or questions within the same conversation. Two works apply CoT, which instructs the model to break its response into multiple steps, such as retrieving disease guidelines and generating recommendations. Only one work utilized few-shot, and 2 works lack sufficient details to classify their strategy. Some works employ multiple prompt engineering strategies, in such cases, they are listed in all relevant categories.

Table 4 presents the prompt engineering strategy used in each work. Works that use zero-shot prompting only provide a single sentence to the model, such as “*How to treat disease?*” in Wilhelm et al. [23], where “disease” is one of the 60 diseases studied in their work, other works follow a similar approach without additional context [22, 25, 55]. The remaining works in this category also use zero-shot with context in addition to zero-shot only prompts [26, 27, 37].

**Table 4:** A general classification of prompt engineering strategies in GenAI-based treatment recommendations

Prompt Engineering	Works
Zero-shot	[22, 23, 25–27, 37, 55, 57]
Zero-shot with context	[12, 15, 16, 29–32, 34, 35, 37]
	[39–43, 45–47, 50–54, 56, 58, 59]
Zero-shot with context	[17, 33, 38, 48, 49]
and follow-up interactions	
CoT	[28, 36]
Insufficient information	[24, 44]

The biggest category, zero-shot with context, includes 27 works that provide a prompt of up to a paragraph long to the model, containing a question and context about the disease or patient symptoms (Table 4). The majority of the works in this category use fictional patient data written by a medical professional as context. Some other works include highly detailed fictional patient data, such as symptoms, demographics, medical tests, and previous comorbidities, often spanning several paragraphs [12, 45]. Other works use less detailed but real patient data, with text only inputs [15, 16, 35, 47, 51]. However, Antaki et al. [15] use images from the Optical Coherence Tomography (OCT) dataset as context, in addition to textual instructions given to a vision-language model.

The two works employed CoT prompting, breaking down the model’s answer into several steps. Lammert et al. [36] design a more sophisticated prompt that follows four consecutive steps: summarization of relevant literature, identification of applicable clinical trials, retrieval of oncology guidelines, and prioritization and synthesis of treatment recommendations. Lafqih et al. [28] engineers the CoT into 5 steps for nutritional management recommendations to: estimate patient’s energy needs, propose macronutrient distribution, provide culturally appropriate meal plans, and provide specific recommendations.

Two works do not provide sufficient information on the prompt engineering strategy used, they report the use of a GenAI model that receive clinical data and generate recommendations, but do not report the exact instructions given to the model to generate treatment recommendations [24, 44].

In summary, prompt engineering is crucial to refine the model’s outputs. Most works rely on some variation of zero-shot prompting, with varying levels of detail from simple queries to more elaborate contextual instructions. The effectiveness of these strategies remains dependent on the model’s parametric knowledge and the quality, clarity, and structure of the prompts themselves.

### (RQ5) How are Datasets used for GenAI Training

We analyze how datasets are used in the selected works, with a focus on their characteristics and suitability for fine-tuning tasks such as SFT, Instruction tuning, CLM, and Question Answering (Q&A). The quality, size, and diversity of the fine-tuning data are key factors in adapting pre-trained models to specialized domains such as TCM [16, 17], management of Diabetes [44], or healthcare in general [50], ultimately improving task-specific performance. Table 5 presents the datasets used for fine-tuning in each work with more than 1000 samples. Column “Real” indicates whether the data used is real (yes), a mix of real and synthetic data generated by AI (both), or synthetic only (No).

**Table 5:** Datasets with more than 1000 samples used for fine-tuning in the selected studies

Task	Lang	Records	Real	Description	Diseases	Source	Availab.	Preprocessing	Work
CLM	CH	1.1B	Yes	General TCM data	Unspecified	Medical records, open source dataset	Authors’ GitHub	OCR, proofreading, label removal	[16]
CLM*	CH	304M**	Yes	General TCM data	Unspecified	Ancient TCM books, public datasets	Authors’ GitHub	Translation to modern CH	[17]
CLM*	EN	1,020	Yes	Medical records, and tomography diagnostic reports	Chronic obstructive pulmonary disease	Affiliated Hospital of Dalian Medical University	Private	Anonymized	[52]
CLM	EN	744,000	Yes	Abstracts, textbooks, articles	Ophthalmo-logy related	PubMed, textbooks, wikipedia	Hugging face: EYE-lit	Remove stop words, chunking paragraphs	[46]
SFT	CH	39,122	Yes	Symptoms with TCM prescriptions	Stomach diseases	Guang’anmen Hospital outpatient records	Authors’ GitHub	Formatted into instructions, data augmentation	[17]
SFT	EN, CH	371,763	Yes	Clinical data with management advice	Diabetes	Shanghai Sixth Hospital, Huadong Sanatorium	Upon request	Anonymized	[44]
SFT	EN, CH	20,000	Both	Clinical data with treatment plans	Acupuncture-related	Unspecified hospital, GPT-3.5, acupuncture guides	Upon request	Filtered and anonymized	[51]
Instruct	CH	3,906	Both	Consultation records	Multiple diseases	National guidelines, hospital records, AI-generated	Authors’ GitHub	Data augmentation with ChatGPT, personal info removed	[50]
Q&A	CH	201,599	Both	TCPM dialogues	Unspecified	ChatGPT 3.5, pharmacopoeia, medicine leaflets	Authors’ GitHub	Data augmentation by ChatGPT	[17]
Q&A	CH	610,921	Yes	TCM Q&A pairs	Unspecified	Encyclopaedia of TCM, public dataset	Authors’ GitHub	Restructured into templates	[16]
Q&A	EN	6,950	No	Psychotherapy QAs	Unspecified	Created by therapists	Authors’ GitHub	No	[60]
Q&A	EN	34,500	Both	A blend of ophthalmic datasets	Unspecified	PMC_LLaMA_ instructions, Icliniq, HealthCareMagic, MedQuad, synthetic dialogues and QAs	Hugging face: EYE-QA+	Data mixing	[46]

*Inferred from data description, **In tokens.

Lang: Language, Availb.: Availability, CLM: Causal Language Modeling, SFT: Supervised Fine-Tuning, Instruct: Instruction-following, Q&A: Question and Answer CH: Chinese, EN: English, TCM: Traditional Chinese Medicine, TCPM: Traditional Chinese Patent Medicine

A key observation is that larger and more diverse datasets tend to yield better results, particularly when combined from multiple sources. For instance, Hua et al. [17] and Dai et al. [16] perform multiple fine-tunings using distinct datasets, combining public and synthesized sources. Another example is Haghighi et al. [60] which gather a diverse amount of ophthalmic data from 4 public datasets and a set of dialogues and Q&As generated with GPT-3.5. The strategy introduces specialized distributions and types of knowledge during training, which can improve generalization and robustness for the intended use [17]. This illustrates how using multiple fine-tuning rounds can systematically inject different knowledge types and improve downstream performance.

The largest dataset reported is the one used by Dai et al. [16], with around 1 billion records aggregated from diverse public TCM sources, used for fine-tuning a model with CLM. In addition, the authors also use another dataset for fine-tuning, composed of Q&A pairs, to further refine the model’s responses for the intended use. They present a strong example of using large datasets that are carefully curated and preprocessed to ensure quality, before fine-tuning an open-source chat LLM [16]. The authors provide the codes and data used in the work in their GitHub. This case exemplifies best practices in transparency, reproducibility, and large-scale data use in fine-tuning.

Another large dataset was used by Hua et al. [17], consisting of approximately 304 million tokens of general TCM content sourced from ancient texts and public datasets. The data underwent translation into modern Chinese before being used for fine-tuning. Additionally, the authors incorporated a smaller, standard supervised fine-tuning dataset of 39,122 real outpatient records from Guang’anmen Hospital. This dataset focused on stomach-related conditions and included data augmentation, aiming to improve the model’s ability to generate relevant prescriptions. The authors also use a third dataset, consisting of questions and answers about traditional Chinese patent medicine (TCPM), with both real and synthetic data. All data and code are open source on the authors’ GitHub. On the other hand the smallest dataset used for training present in Table 5 is composed of 1,020 patient records, due to the rarity of the disease [52].

Li et al. [44] and Li et al. [51] worked with clinical datasets in both Chinese and English, targeting diabetes and acupuncture-related conditions, respectively (Table 5). The former included 371,763 records with anonymized patient management data from two hospitals, while the latter compiled 20,000 records from an unspecified hospital and included additional data generated by GPT-3.5 and acupuncture guides. These datasets were filtered, anonymized, and used in supervised fine-tuning tasks to enhance clinical decision-making capabilities. The datasets used for fine-tuning in the reviewed works, presented in Table 5, contain more than 1000 samples, however one work used datasets of 300, 593, and 146 samples for fine-tuning models for psychotherapy intervention, in addition to another fine-tuning with a dataset of 6,950 samples of therapy Q&As crafted by therapists [60]. Across the studies that fine-tuned their models, a trend emerges where multi-source datasets, careful preprocessing, and targeted augmentation contribute significantly to model adaptation.

Several works used datasets to evaluate LLMs. These include smaller datasets for assessing general-purpose LLMs, a detailed description of these datasets is presented in Supplementary Material 1. Examples include Truhn et al. [47], who used 20 MRI textual reports, and Ghersin et al. [31], who used 30 patient cases, both works evaluate GPT-4. The works by Benary et al. [37] and Lammert et al. [36] used the same 10 fictional lung cancer patient cases to evaluate several general-purpose third-party LLMs. The latter also used a dataset with oncology-related guidelines for RAG (see Supplementary Material 1).

This variety in dataset type and size reveals that evaluation methods remain exploratory and lack standardization across studies. As new models emerge, an initial evaluation of their capabilities in medical domains can serve as a first filter when selecting models, since those that already perform well in such domains can be leveraged for further fine-tuning, improving their results even more.

The largest dataset used for evaluation was by Iqbal et al. [22], with 12 million real patient records covering various skin diseases, to evaluate GPT-4. In contrast, Fisch et al. [33] used only a single record to evaluate treatment recommendations for bacterial meningitis by models from Google, Microsoft, OpenAI, and Meta. The authors acknowledge that “the study was limited to a single case scenario, and the results may not be generalizable to other clinical scenarios” [33]. Lee et al. [25] used 36 clinical prompts, and Huo et al. [30] used 100 surgical consultation records to evaluate general-purpose pre-trained models. This wide range of evaluation dataset sizes suggests the need for dedicated medical benchmarks that balance realism, diversity, and scalability, to evaluate models [59].

In Supplementary Material 1, we present the only example of a multimodal dataset using both text and images. Antaki et al. [15] used a public dataset of OCT retinal scans combined with a standard textual prompt written by the authors in Gemini Pro, which processes both images and text. Similarly, Li et al. [44] also used text and retinal images, however, the images were part of a separate model which, when combined with an LLM, forms a tool that can suggest treatments based on patient data to the medical professional. Notably, patients preferred consultations where physicians were assisted by AI.

Preprocessing to improve data quality is crucial for ensuring GenAI model performance, as data quality directly impacts results. We highlight the work of Liu et al. [53], who present a methodology for data selection and preprocessing. They used a search string to screen 306 medical research papers from ScienceDirect, Web of Science, and PubMed, excluding non-English articles, case reports, reviews, brief communications, and studies with sample sizes below 100. Only peer-reviewed papers published within the last five years with at least five citations were included. Non-essential sections, such as references and acknowledgments, were removed using regular expressions and reviewed by a medical expert. The processed texts were then converted into plain text files to fit into ChatGPT’s context window.

Other works have focused on diverse preprocessing and dataset curation techniques to enhance model reliability. For instance, Truhn et al. [47] anonymized MRI reports by removing personal information and modifying history sections to eliminate biases in treatment recommendations. Zakka et al. [54] optimized RAG efficiency by segmenting articles into 1000-token chunks and encoding them into high-dimensional vectors, ensuring that only the most relevant information was used for response generation that fits in the model’s context window. Meanwhile, Liu et al. [50] curated a dataset of Chinese patent medicine instructions, employing rigorous cleaning, annotation, and feature extraction techniques. These efforts demonstrate the range of preprocessing strategies applied to medical datasets, from standardization and anonymization to advanced retrieval and data augmentation techniques.

Some works use smaller datasets, with 36 clinical questions as input for the GenAI models [25]. Huo et al. [30] uses 100 records, of which 60 are clinical cases aimed at surgeon consultations, and 40 are simulated questions formulated for patients. Other examples include the study by Liu et al. [53], which used 241 records extracted from medical research articles, and Zakka et al. [54], which uses 314 clinical questions organized by experts.

The differences in the volume and nature of the records reflect the variety and lack of standardization in methodological approaches adopted by the authors. It is important to note that the quantity and quality of records can directly influence the generative capacity of GenAI models, especially in the context of training, where large volumes of curated data tend to improve the quality of generated responses [8]. Additionally, when using patient records for evaluation, the amount and diversity of data can significantly impact the reliability of results, evaluating LLMs with data from a single hospital might limit the generalizability, making it less reliable to use the models in different clinical settings [43, 58].

We observe emerging trends such as the combination of real and synthetic data, multimodal inputs, and diverse preprocessing techniques tailored to specific domains. Altogether, these findings emphasize that both dataset size and thoughtful design, including source integration, curation, and preparation, are critical to maximizing the performance, reliability, and applicability of GenAI in healthcare.

### (RQ6) How are GenAI Models Evaluated for Treatment Recommendations?

The evaluation of GenAI models requires a comprehensive assessment that considers both quantitative and qualitative metrics. While quantitative metrics provide objective, numerical measures of model performance, qualitative metrics assess the appropriateness, interpretability, and factuality of AI-generated recommendations.

Quantitative metrics focus on measuring performance through statistical and mathematical analysis. These include classification-based metrics such as F1-score, precision, recall, and specificity, which evaluate how accurately a model distinguishes between correct and incorrect medical recommendations [15, 17]. Similarity-based metrics, such as cosine similarity [36] and sentence similarity scores such as BLEU and ROUGE [50], assess how closely AI-generated recommendations align with expert references. Additionally, statistical tests, including ANOVA, chi-square, and correlation coefficients, are used to compare AI-generated outputs with human decisions, ensuring consistency and reliability.

Qualitative metrics emphasize the interpretability and appropriateness of GenAI outputs. These include readability scores (to measure the ease of understanding AI-generated text) [25], factuality assessments (to ensure medical accuracy) [54], and expert evaluations (to gauge clinical relevance) [22]. Furthermore, aspects like coherence, completeness, and citation quality are crucial for determining whether an LLM provides well-structured and evidence-based recommendations [26].

Specialized metrics assess GenAI performance in specific medical fields. Examples include diagnostic accuracy (TI-RADS classifications) for radiology, appropriateness of treatment per subspecialty, and subjective feedback from medical experts. Table 6 summarizes the evaluation metrics used in the selected works.

**Table 6:** Evaluation metrics used in the selected works

Category	Metrics	Works
Quantitative	Cosine Similarity	[36, 57]
	Precision	[16, 17, 43, 60]
	Recall	[15–17, 43, 52, 59, 60]
	Accuracy	[16, 17, 24, 27, 30–32, 35, 37, 38, 44, 45, 48, 52, 53, 60]
	F1 Score	[15–17, 43]
	Specificity	[15, 52, 59]
	False Positive Rate	[59]
	Negative Predicted Value	[59]
	Statistical Analysis	[15, 25, 31, 37, 45, 49]
	Fleiss’ Kappa	[45]
	Cohen’s Kappa	[12, 42, 43, 45]
	ANOVA	[15, 37, 45]
	Chi-square tests	[15]
	Student’s t-test	[37]
	Inter-Rater Agreement	[15, 31]
	Pearson correlation coefficient	[23, 38]
	Average reliability	[38]
	The sentence similarity evaluation	[51]
	BARTScore	[46, 50]
	BERTScore	[16, 46, 56]
	BLEU	[16, 46, 50]
	ROUGE	[16, 50, 56]
	Time Efficiency	[53]
	Q-Value	[22]
	METEOR	[16]
	MRR	[16]
	nDCG	[16]
	MAE	[16]
	RMSE	[16]
	ROC-AUC	[16]
	RAGAS	[34, 56]
	TrueLens	[56]
	Word count	[56]
Qualitative	Likert Scales	[28, 29, 42, 46, 47, 49, 55, 56, 58]
	Consistency	[33, 47]
	Readability assessment	[25, 32]
	Appropriateness of responses	[25, 32, 44]
	Evaluations from experts	[15, 17, 22, 33–37, 43–46, 48, 53, 57]
	Report structure	[35]
	Factuality	[54]
	Completeness (0-1)	[54]
	Completeness (binary)	[57]
	Preference	[54]
	Citation Quality	[54]
	Adversarial Safety	[54]
	Communication	[26]
	(Correct, Concise, Comprehensive, Comprehensible)	
	Truthfulness	[23]
	Harmfulness	[23]
	mDISCERN score	[23]
	Clinical recommendation	[39–41]
	Explanation	[39–41]
	Summarization	[39–41]
	Analytical Hierarchy Process	[28]
	Risk Matrix Analysis	[29]

One study evaluated a customized ChatGPT model for surgical treatment recommendations for GERD, comparing it to the generic ChatGPT-4 model. The customized model, referred to as GTS, delivered accurate recommendations in 100% of both surgeon (60/60) and patient (40/40) consultations, consistently citing personalized clinical guidelines. In contrast, the generic ChatGPT-4 model demonstrated lower accuracy, 66.7% for surgeons and 47.5% for patients and failed to provide supporting evidence in its responses [30]. This highlighted the potential of customizing LLMs with specific system prompts to enhance precision and trustworthiness in clinical recommendations.

In the reviewed studies, a clear emphasis was placed on performance evaluation using metrics such as accuracy and precision [24, 27, 30]. While accuracy remains a widely used metric due to its intuitive appeal, it may be insufficient in scenarios with imbalanced datasets. In such cases, metrics like the F1-score become critical for a more balanced evaluation of model performance as were used in some of the works [15–17]. The integration of multiple metrics enables a more comprehensive assessment, increasing confidence in the applicability of GenAI systems across diverse real-world clinical scenarios. Nonetheless, the need for expert oversight in interpreting outputs remains a consistent theme in the literature [35, 37, 45].

The literature reveals a lack of consistency in how GenAI treatment recommendations are evaluated. Many studies develop their own custom metrics tailored to specific use cases, reflecting the lack of clarity when it comes to evaluation. Two studies even create a similar metric to analyze the completeness of the answer but one is continuous while the other is binary [54, 57]. This stands in contrast to more established areas like machine learning classification, where a core set of metrics, such as accuracy and precision, is consistently applied. The absence of dedicated and standardized benchmarks in this emerging field also poses challenges for comparing results across studies and highlights the ongoing need for shared evaluation frameworks [59].

### (RQ7) What Ethical and Regulatory Considerations arise from the use of GenAI in Medical Treatment Recommendations?

Most of the selected works express concerns about the use of GenAI in medical treatment recommendations. These concerns range from minor issues to serious problems that hinder the use of GenAI in real-world settings. In this section, we synthesize some of the ethical and regulatory issues reported by the selected works.

The use of GenAI in medical treatment recommendations raises critical concerns, especially with respect to accuracy, reliability, and potential risks to patients. A major issue shared among the works is the risk of inaccurate recommendations. This issue can arise due to input quality, lack of patient-specific context, and the potential for patient misuse [47]. Additionally, the non-deterministic nature of these models may produce hallucinations by generating incorrect or fabricated information, raising concerns about the reliability of the generated recommendations [54]. GenAI models can also generate recommendations that lack evidentiary support and may be inconsistent with clinical guidelines [30].

The variability in responses and the absence of transparency in GenAI’s reasoning process pose additional risks, making it difficult for users to assess the validity of its outputs [33]. Ensuring the accuracy and reliability of treatment recommendations remains the primary concern [27, 44]. The potential for incorrect treatment recommendations and unreliable information raises significant challenges regarding the role of GenAI in healthcare. In surgical decision-making, accuracy becomes even more critical, as errors could propagate misinformation and lead to unsafe medical interventions [32]. In fields such as oncology, errors in interpreting tumor profiles could lead to incorrect treatment plans, further emphasizing the need for caution [37].

Even when models produce recommendations that seem safe and accurate, they often fail to capture essential details that may not be present in the training data or the input provided, increasing the likelihood of incorrect treatment recommendations [49, 55]. The confidence with which these models generate responses can be misleading, inducing trust in possibly incorrect information. For instance, Nabieva et al. [38] expressed concerns that in complex medical topics, the model can generate highly confident yet incorrect information. Similarly, Zeng et al. [55] also reports that the models can generate seemingly correct information with wrong details that are hard to detect, specially in complex cases, and Shrestha et al. [48] noted that ChatGPT may confidently provide incorrect responses.

In addition to accuracy, concerns also arise regarding the potential to reproduce biases and inequalities present in training data, which may disproportionately affect different patient populations and medical professionals. For instance, Lautrup et al. [26] report that ChatGPT might generate dangerous or nonsensical responses, particularly affecting users with low medical literacy, which may lead to self-diagnosis or undue influence on medical professionals. In contrast, users with more medical knowledge can provide more informed inputs, leading to better response quality. Even when using carefully curated medical data from trusted sources, biases can be reproduced in the models responses, and when the data is generated by another model it can also pass along biases [56].

Biases in training data also introduce risks related to compliance with regulatory and legal standards. Zakka et al. [54] warned that biased outputs may reinforce gender, racial, or religious stereotypes, underscoring the importance of safeguarding patient privacy and adhering to healthcare regulations. Ensuring accuracy, transparency, fairness, and compliance with established medical standards is essential to maintaining trust in AI-generated recommendations [50].

The lack of transparency exacerbates the challenges, proprietary and paywalled datasets limit oversight, raise patient data privacy concerns, and hinder the development of explainable AI models [36]. The reliance on a small number of closed corporations that control data and training of the highest performing models limit transparency [57].

Additionally, biases stemming from training on specific populations emphasize the need for diverse, unbiased datasets and well-defined ethical and legal frameworks for safe implementation [44]. The opacity of data sources further complicates accountability, increasing the risk of exposing sensitive information to misuse or unauthorized access [12]. In some cases, such as LLM-generated prescriptions, AI-driven recommendations lack proper medical validation and remain unapproved for clinical use, increasing concerns about their safety and reliability [17].

To deploy GenAI models in real clinical settings, rigorous regulatory oversight is required to ensure both safe and ethical use [54]. Currently, models such as GPT-4 are not approved as medical tools, necessitating transparency, ongoing monitoring, and user education to mitigate risks [47]. Despite the growing integration of AI into healthcare, clear regulatory frameworks for approving GenAI clinical decision support tools remain lacking, particularly in specialized fields such as gastroenterology [31].

A major regulatory challenge is the lack of explainability in GenAI models, which, combined with the risk of incorrect treatment plans, hinders regulatory approval [45]. Obtaining approval remains a key concern in medical decision-making, as regulatory uncertainty and transparency issues persist [32]. Strict regulations and comprehensive validation are essential before these AI tools can be safely integrated into clinical practice [15]. Legal liability is another concern, as AI-generated recommendations raise questions about accountability, who is legally responsible for a recommendation that causes harm to a patient remains undefined [25, 42]. To mitigate these risks, regular assessments, oversight, and fine-tuning are necessary to ensure clinical safety [23]. Li et al. [51] further emphasized the need for strict regulatory measures before these models can be applied in clinical settings.

Beyond regulatory challenges, ethical considerations also play a crucial role in the responsible deployment of GenAI in healthcare. Many works reported received approval from an ethical commttee. For instance, Wang et al. [35] reported that their study adhered to the Declaration of Helsinki and received ethics committee approval, noting that informed consent was not required since no human subjects were directly involved. However, other studies did not explore ethical or regulatory considerations in depth. Papastratis et al. [24] and Iqbal et al. [22] provided limited discussion on these aspects, while Dai et al. [16] and Liu et al. [53] similarly did not explicitly address ethical or regulatory concerns related to the use of GenAI in medical treatment recommendations.

As the integration of GenAI into medical treatment recommendations continues to evolve, addressing ethical and regulatory concerns remains imperative. While most studies acknowledge the need for oversight, transparency, and compliance with ethical standards, some fail to engage with these critical issues. One clear pattern arise, studies report the need for clearer regulatory frameworks and standardized ethical guidelines to ensure the safe and responsible deployment of GenAI in clinical settings, highlighting that this is still an open issue.

### (RQ8) What are the common Challenges and Limitations Associated with the Implementation of GenAI in Clinical Practice?

The integration of GenAI into clinical practice presents several challenges and limitations that slow down its widespread adoption and effectiveness. Based on the reviewed literature, the most frequently cited issues relate to reliability, bias and ethics, transparency, scalability, computational costs, regulatory concerns, and performance in complex scenarios. To summarize these challenges, Table 7 presents a structured overview of the key challenges associated with the implementation of GenAI in clinical practice, along with the corresponding works that have explored these issues.

**Table 7:** Challenges and Limitations of GenAI in Clinical Practice Reported by the Works

Challenge	Description	Works	Count
Reliability Issues	Hallucinations, generic responses, inconsistencies, data dependency	[15, 22, 23, 27, 29, 31–36, 38–43, 46–49, 52, 54, 55, 57–59]	28
Bias and Ethics	Racial/gender biases, dehumanization of care, disparities	[22, 30, 44, 46, 55, 56, 59]	7
Transparency and Interpretability	“Black box” nature, lack of justification, difficulty in validation	[12, 23, 25, 32, 35, 45, 48, 51, 57]	9
Technical and Scalability Challenges	Model updating, EHR integration, generalization	[17, 30, 32, 37, 50, 53, 54]	7
Computational Costs	GPU resources, training time, scalability	[16, 17, 50]	3
Regulation and Privacy	Compliance, data protection, regulatory gaps	[12, 24, 27, 30, 32, 34, 44, 53–55, 59]	11
Difficulty in Complex Scenarios	Rare cases, nuanced reasoning, adaptation to specific contexts	[15, 23, 28, 29, 31, 33–42, 47, 49, 51, 53, 55, 57]	22

One of the predominant concerns is reliability, as GenAI models are susceptible to generating hallucinations, inconsistencies, and overly generic responses, this is mentioned in 28 studies (Table 7). These issues arise from the data-dependent nature of these models, which can lead to incorrect or misleading medical advice [47]. Given that clinical decision-making relies on accuracy and precision, this limitation raises significant concerns regarding patient safety.

Another major challenge is bias and ethical concerns, particularly with respect to racial and gender biases that may arise due to biases already present in the training data [22]. Additionally, the dehumanization of care is a growing concern, as the overreliance on AI-based decision-making could reduce the empathetic and personalized aspects of healthcare [22, 30, 44]. Disparities in AI-generated recommendations have been noted, which could exacerbate existing inequalities in healthcare access and treatment outcomes [26].

The lack of transparency and interpretability is another pressing issue. Many GenAI models function as “black boxes,” meaning that their decision-making processes are opaque and difficult to validate [35]. This lack of interpretability makes it challenging for clinicians to trust AI-generated outputs, especially in high-stakes environments where accountability is crucial.

In terms of technical and scalability challenges, the integration of GenAI with existing Electronic Health Records (EHRs) remains a major bottleneck [30, 54]. Generalizability is also a significant challenge, single-center studies might not represent the full scope of the models’ results [58]. Additionally, continuous updates are required to ensure that AI models remain relevant and aligned with evolving medical guidelines.

Computational costs further complicate the widespread adoption of GenAI in clinical settings. The high demand for GPU resources, extended training times, and scalability issues pose economic and logistical barriers, particularly for resource-limited environments [16, 17, 50]. Without significant advancements in efficiency, the cost of implementing and maintaining these AI systems may outweigh their perceived benefits.

Regulatory and privacy challenges represent another crucial barrier. The use of AI in clinical practice must comply with data protection laws, ethical standards, and evolving regulatory requirements [24, 30, 54]. The absence of standardized regulations and guidelines for AI-driven healthcare applications creates uncertainty, making it difficult for institutions to fully integrate these technologies into clinical workflows.

Finally, GenAI models face difficulty in handling complex clinical scenarios. Many AI systems struggle with rare diseases, nuanced reasoning, and adapting to specific medical contexts, limiting their applicability in specialized domains [23, 31, 35, 47]. These challenges underscore the need for more context-aware and adaptable AI models capable of handling the intricacies of real-world medical decision-making.

### (RQ9) What Future Research Directions are Suggested by Current Works on GenAI for Treatment Recommendation?

**Fig. 5:**
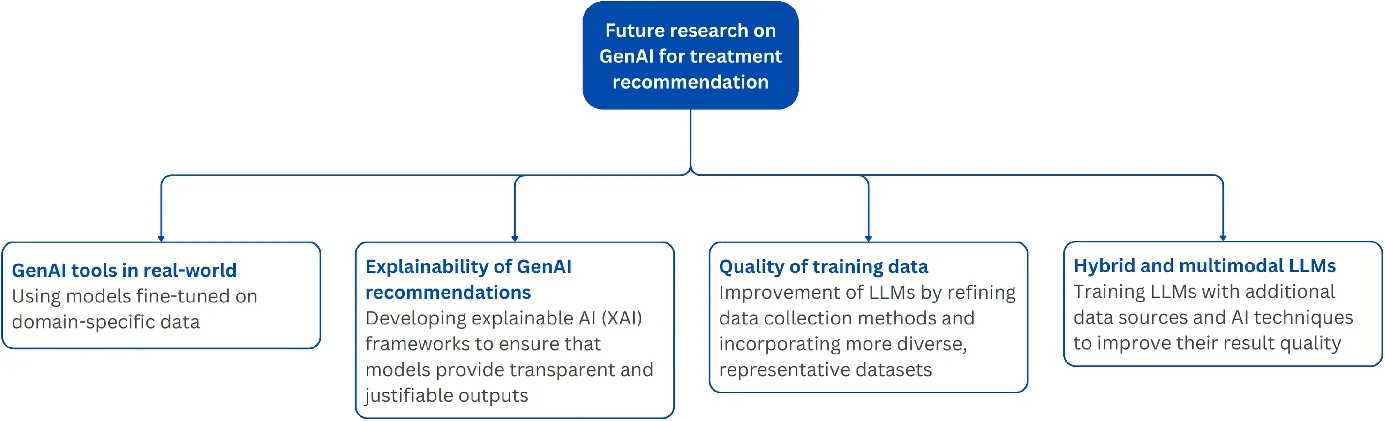
Future works found in literature

Future research directions suggested by the current works often focus on addressing barriers that prevent the acceptance of GenAI tools in real-world clinical settings. Many of these challenges come from concerns about the risks posed to patients when relying on AI-generated treatment recommendations. To mitigate these risks, future directions include the use of models fine-tuned on domain-specific data, as presented in Fig. 5.

While generalist models have demonstrated success in natural language tasks, they may not be well-suited for specific medical applications without proper fine-tuning. In fact, developing domain-specific models tailored to particular medical fields is a significant research focus urged by the selected works, specially open weights models for local use in clinical settings [59]. Several works highlight the need for fine-tuning AI models with specialized datasets, such as cancer-related data [37], retinal images [15], and bariatric surgery-focused AI systems [25]. Other works emphasize the need to expand the use of LLMs to support TCM by integrating specialized knowledge bases and refining domain-specific adaptations [16, 51].

Before the implementation in real-world, GenAI model validation in clinical environments remains a crucial step for adoption. Several works suggest extensive clinical trials, prospective validation, and usability studies to assess AI reliability and effectiveness in practice [15, 30, 33, 36]. Works also suggest evaluating the impact of medical decision-making process enhanced with AI-generated recommendations in clinical workflows [32, 37].

Another critical challenge is improving the explainability of AI-generated recommendations to enhance trust and usability. Multiple works stress the importance of developing explainable AI (XAI) frameworks such as SHAP [56], to ensure that models provide transparent and justifiable outputs [12, 17, 31, 50]. Some works mention that efforts to improve interpretability are especially relevant in TCM [16, 17, 50, 51].

The quality of training data and bias mitigation are also pressing concerns. Research suggests improving LLMs by refining data collection methods and incorporating more diverse, representative datasets [44, 50, 54]. Addressing biases in medical AI models is particularly critical for ensuring equitable healthcare outcomes, as real-world data may contain biases that affect model responses [27].

Finally, hybrid and multimodal approaches are gaining attention, integrating LLMs with additional data sources and AI techniques to improve result quality. Studies propose combining vision-language models with clinical data to enhance diagnostic capabilities for cases where medical images are important [35, 45, 47]. Other suggestions include leveraging reinforcement learning with human feedback for better model refinement and alignment with human preferences [23, 54], incorporating real-time patient monitoring via wearables [44], and smart home devices [59] to enhance AI-driven decision-making.

Due to various barriers preventing LLMs from being a reliable tool for clinical decision support, most studies remain theoretical or evaluative. Implementing these models in clinical practice is particularly challenging in such a critical field as healthcare. The current state of the art is still in its early stages, focusing on methods to improve the reliability of LLMs. As a result, much of the research highlights concerns about model reliability and explores potential areas for improvement.

With little to no real-world adoption in clinical settings in the reviewed works, LLM applications still have a long way to go before becoming viable. The suggested future directions consistently emphasize the need for further research to enhance model reliability and safety before LLMs can be effectively used for clinical decision support.

## Discussion

Most works use third-party general-purpose models, post-trained on instructional datasets to follow the user’s intent as virtual assistants. We found that models such as OpenAI’s GPT-3 and GPT-4 [24, 25, 32, 33, 45, 51, 53], Claude by Anthropic [15, 23, 33], Gemini Pro by Google [15], and Llama by Meta [36, 44, 63] are frequently used in the reviewed works without any fine-tuning on medical data. However, because these models are designed for general-purpose applications, they may not always produce optimal results in medical contexts [18].

To address this limitation, researchers often turn to prompt engineering as a means of improving response quality. The prompt engineering strategies used in the selected works are often a variation of zero-shot, which is the simplest strategy [8]. The findings indicate that detailed and technical prompts, with the addition of context and patient data, generally enhance the quality and relevance of generated recommendations [26]. The quality of responses often reflects the quality of the prompts, which can intensify existing inequalities among populations that are less proficient at formulating prompts. Users with less knowledge about prompt engineering are more likely to receive lower-quality responses [26]. However, even with detailed context, zero-shot approaches remain dependent on the model’s internal knowledge and the quality of its pre-training and post-training, which may not always align with medical best practices. This raises questions about the use of general-purpose models for medical decision support, suggesting that models fine-tuned on medical data could be a better alternative [16, 17, 50]. The widespread reliance on zero-shot strategies highlights a limited adoption of more advanced prompt engineering techniques, such as CoT, which has been shown to improve response quality [8, 36].

A notable gap in the literature is the limited number of works that study the generation of treatment recommendations using real patient data. Most works rely on synthetic cases, often created by medical professionals, which may not fully capture the variability and complexity of real-world clinical scenarios. The hesitancy in using real patient data could be attributed to concerns about data privacy, ethical considerations, and the challenge of ensuring reproducibility in GenAI-generated recommendations [26]. Furthermore, the absence of standardized best practices for prompt engineering in medical applications may contribute to inconsistencies across works.

General-purpose models are not well-suited for most medical applications, as mentioned before. Customizing a GPT-4 model on specialized, curated medical data yields higher-quality results [30]. Further fine-tuning a model on TCM and TCPM yields better quality results [16, 50]. The selected works support that adapting models to domain knowledge improves response quality [51].

A recipe for high-quality medical GenAI models involves using the largest full-precision (no quantization) state-of-the-art foundation model possible. The model should be trained on the entire available internet data to acquire language understanding capabilities, fine-tuned on instructional data (instruct/assistant models), and further fine-tuned on medical data. Full-weight tuning is not necessary; techniques such as parameter efficient fine-tuning (PEFT) or low-rank adaptation (LoRa) can be used [17]. Additionally, RAG and prompt engineering should be incorporated to enhance performance [36]. The model should be parametrized to define parameters for manipulating the model’s output logits, such as temperature, top-p, and top-k, using validation data to assess its performance. Techniques can be used to automate the parametrization process. The model should be used as a tool to assist medical professionals, the final medical decision should always be up to the professional

In summary, the literature consistently supports that the best practice for improving disease treatment recommendations is to infuse models with as much domain-specific knowledge as possible. This involves training on large, high-quality medical datasets, ideally using techniques like traditional fine-tuning, PEFT, or LoRa [17], in combination with robust prompt engineering and retrieval strategies such as RAG [36]. Such specialization significantly enhances the accuracy, reliability, and clinical relevance of model outputs, reinforcing the importance of tailoring GenAI systems to the medical domain.

## Conclusions and Future Directions

This study has systematically explored the application of GenAI models in providing treatment recommendations for various diseases, highlighting both the advances and the existing research gaps. Although GenAI technologies demonstrate potential for optimizing clinical decision-making and personalizing patient care, significant barriers remain, such as limited real-world validation, risks of bias, and regulatory uncertainties. Furthermore, most research efforts have focused on general-purpose models lacking fine-tuning on specialized medical data.

Future research should therefore address two main challenges: (a) refining model training and evaluation with high-quality clinical data, aiming to enhance both safety and domain-specific relevance, and (b) conducting robust clinical studies to ensure the efficacy and reliability of AI-generated recommendations. Ultimately, wide adoption of GenAI in healthcare settings demands a multidisciplinary effort involving researchers, healthcare professionals, ethics experts, and regulatory bodies. This collaboration is essential for developing advanced solutions that not only broaden the benefits of AI in patient care but also safeguard against the inherent risks of its use.

## Supplementary Information

This article has accompanying supplementary files: Supplementary Materials 1 and 2.

## Supplementary Information

Below is the link to the electronic supplementary material.

Supplementary file 1 (zip 583 KB)

## Data Availability

No datasets were generated or analysed during the current study.
